# When conflict influences liking: The case of the Stroop task

**DOI:** 10.1371/journal.pone.0199700

**Published:** 2018-07-11

**Authors:** Tom G. E. Damen, Madelijn Strick, Toon W. Taris, Henk Aarts

**Affiliations:** Department of Psychology, Utrecht University, Utrecht, The Netherlands; University of Melbourne, AUSTRALIA

## Abstract

Research suggests that cognitive conflict is accompanied by a negative signal. Building on the demonstrated role of negative affect in attitude formation and change, the present research investigated whether the experience of cognitive conflict negatively influences subsequent evaluations of neutral stimuli. Relying on the emergence of conflict in the Stroop task, participants were presented with compatible (non-conflict) and incompatible (conflict) Stroop color words that were each followed by a neutral visual stimulus. In general, participants liked stimuli following incompatible Stroop words less than stimuli following compatible Stroop words. The results revealed similar compatibility effects in tasks in which participants actively responded to the Stroop words and in tasks in which they passively observed them. Furthermore, these effects emerged in offline and online measures of evaluation. Interestingly, the results also suggest that the compatibility effect on liking observed in the present research was to some degree driven by the positivity associated with the compatible Stroop words, and not just by the negativity associated with the incompatible Stroop words. We discuss the present findings in the context of how and when conflicting responses to events (such as in the Stroop task) can influence evaluations of stimuli associated with the conflicting events.

## Introduction

In everyday life the word *conflict* is often used to describe a negative event or to refer to a situation of doubt and uncertainty. In the research domain of cognition and behavior, however, the term is used to describe a cognitive difficulty that arises when mental processes are at odds or provide conflicting information. Think for example of the famous Stroop conflict task, in which an individual may find it difficult to indicate that a word is printed in a blue font when that word reads ‘*red*’ [1]. This task poses a challenge because the automatized processes of color perception and reading interfere and provide contradictory information [2]. This is why we often need some time to process and successfully respond to a stimulus that presents conflicting information, such as an incompatible Stroop word.

Cognitive conflicts, like the one’s experienced on the Stroop task, may represent signals that people cognitively experience as negative [3]. The empirical quest to investigate the validity of this conflict negativity assumption has only just begun [4][5], but it has already led to some interesting insights and promising new research questions. For example, if conflict is indeed experienced as negative, such negativity may in turn influence other processes related to affect. Specifically, the absence or presence of conflict could influence ones’ explicit evaluations of associated stimuli. Considering that these conflicts are argued to emerge regularly and across a wide range of domains [6][7][8][9][10][11][12] they may often influence and change our evaluations. It is therefore important to investigate the relation between cognitive conflict and explicit evaluations.

In the current research, we investigated whether the Stroop task, a paradigm of cognitive conflict, could influence a person’s evaluations of associated stimuli. Furthermore, by varying specific methodological parameters in the Stroop conflict task, the present research additionally explored *when* Stroop conflict effectively influences evaluations.

### Conflict monitoring, adaptation, and negativity

When confronted with cognitive conflicts people rely on a system of cognitive control to help them resolve the current conflict they are experiencing. It is argued that when conflict is perceived, a process of cognitive recruitment starts to facilitate the ability to deal with the conflicting situation ([13], but see [14] and [15]). This may be why individuals, after an initial task in which conflict is experienced, subsequently tend to do better on a challenging task. The reduction in cognitive interference effects following incompatible trials reflects a process known as conflict adaptation. Conflict adaptation effects have been intensely studied over the past decade [16] and have been shown to occur on a wide range of tasks, including the Flanker task [17], the Simon task [18], and relevant for the present purpose, the Stroop task [19].

It is argued that conflict adaptation effects occur out of the motivation to resolve the cognitive conflict–which is experienced as a negative signal [3]. This negativity may even be instrumental in recruiting the cognitive control that is necessary for conflict adaptation to occur [20]. Brain imaging studies have suggested that the Anterior Cingulate Cortex (ACC) plays a major role in the detection of cognitive conflict [19][21]. Interestingly, this same area also becomes active when errors and other aversive signals are observed [22][23][24]. Thus, the ACC responds similarly to cognitive conflict and negative affect, possibly registering both as aversive signals [25][26].

Researchers also more directly investigated whether cognitive conflict is indeed processed as a negative signal. In two studies, Dreisbach and Fischer [5] and Fritz and Dreisbach [27] investigated whether individuals’ responses and evaluations were influenced when participants were primed with Stroop words. The Stroop words were merely presented, and participants were not required to respond to them as in a classical Stroop task. Their results showed that participants more easily categorized subsequent negative words as negative [5], and were more likely to assign negative labels to subsequent neutral stimuli [27] when these targets were shortly preceded by incompatible Stroop words. These studies led to three important insights: First, that the cognitive conflict that emerges from the Stroop task is related to negativity. Second, that such negativity emerges relatively automatically. And third, that this negativity even emerges when individuals merely perceive Stroop conflict–in other words, conflict and conflict negativity do not only occur when competing responses are activated (e.g., [20]).

### Prime versus response conflict

The idea that conflict may emerge without a requirement to respond (or without a processing goal) is a relatively new one and requires more empirical support. Furthermore, it is intuitive to explore what happens if one experiences both perceptual/prime conflict *as well as* response conflict—as is the case in the classical Stroop task. Take for example an incompatible Stroop trial. First, there may be the conflict negativity triggered by the stimulus itself: The incompatible Stroop color word triggers two automatic processes providing incompatible information—the automatic process of word reading and the second automatic process of color categorization. A second way in which conflict negativity may then occur is when we experience response interference–it is difficult to inhibit one response in favor of the other–and the more interference one experiences when responding, the more cognitive conflict and conflict negativity may emerge. The question then remains whether response conflict will trigger negativity *above and beyond* the negativity that has been shown to emerge from conflict without a response requirement.

In the present research we will address conflict negativity both in situations in which *there is no* requirement to respond, and in situations in which *there is* a requirement to respond to the conflicting stimuli.

### Stroop conflict–a powerful mechanism to influence evaluations?

Attitude formation and change have often been described along the lines of dual process theories [28][29][30]. Such theories acknowledge that attitudes can emerge through reasoning and deliberation, but also through heuristical processing of information [31][32]. A person’s positive or negative mood state may, for example, influence the way that person evaluates a stimulus in the environment. We therefore often use affective information in our evaluations.

Over the past two decades the literature on Evaluative Conditioning (e.g., [33][34]) has shown that affective information also influences evaluations at a low-level of cognitive processing. Presenting stimuli close in time, thereby creating an association between them, leads to valence transfer between the stimuli. Therefore, when a neutral stimulus (conditioned stimulus, CS) becomes associated with a negative stimulus (unconditioned stimulus, US), the CS is subsequently liked less. EC-theory has become an important model for human cognition and behavior: It explains how people develop likes and dislikes for objects, and how they learn which objects to approach and which objects to avoid.

To establish the evaluative conditioning effect, previous studies typically used USs that were positive or negative due to meaning: People have learned to think of ‘death’ as being something negative and ‘love’ as being something positive. Therefore, when they subsequently hear the word ‘death’ it will negatively influence the evaluation of an associated stimulus. The previously discussed work by Dreisbach and Fischer [5] and Fritz and Dreisbach [27] however shows that the valence of a US is not only determined by meaning, but also by a stimulus’ perceptual features, and whether those features are aligned in a compatible or incompatible manner. Their work suggests that conflict negativity can transfer to *other* stimuli: Stimuli that trigger negativity through their conflicting features can create an EC-like effect. People may therefore not only develop their likes and dislikes through associations with stimuli that are positive and negative in meaning, but also when neutral stimuli trigger processing conflicts. However, before we can convincingly claim that say Stroop conflict is a new and promising way to cause attitude change, more empirical studies are required to replicate and establish this effect.

### Online versus offline evaluations

The studies that explored the nature of cognitive conflict have focused on the online properties of conflict. As discussed above, conflicting events have been shown to speed-up responses to negative (versus positive) stimuli and bias the negative categorization of neutral stimuli [5][27]. As these findings suggest that conflict is experienced as a negative and aversive event, the use of online measures to capture the hedonic properties of conflict is an appropriate approach when one aims to examine the immediate (trial-by-trial) affective priming effects of cognitive conflict. However, exposure to conflict can occur repeatedly and frequently, and evaluations of stimuli associated with the conflicting event cannot always be explicitly elicited and assessed online on a trial-by-trial basis. A person may make an evaluative judgment about a stimulus or event after some delay (offline), or, after the stimulus/event has already been repeatedly paired with multiple instances of cognitive conflict. It is therefore interesting to explore whether cognitive conflict also influences offline evaluations.

Importantly, differences between on and offline measures may well emerge. For example, online evaluations rely on processing goals. Such goals can guide information processing and attention before conflict and co-occurring stimuli are perceived (cf. [35]). However, participants may be less inclined to attend to conflicts and the evaluation of associated stimuli when such processing goals are absent, and evaluations are delayed and therefore memory-based (see e.g., [36],[37]). While Stroop conflict has been shown to affect online and immediate affective categorization of stimuli [27], the question therefore remains whether the repeated pairing of conflict with specific stimuli will also modulate offline and explicitly assessed liking ratings of those stimuli. The present research set out to explore this issue further.

### Conflict negativity versus non-conflict positivity

Until now we have assumed that Stroop conflicts are experienced as negative events and that the negativity associated with conflict could influence evaluations. This assumption comes naturally considering that the research on conflict adaptation emphasizes the negativity of conflict as the driving force behind conflict detection and adaptation (e.g., [2][20]). However, it is also possible that incompatible stimuli (or stimuli associated with incompatibility) are not only liked less, but that compatible stimuli are liked more. This is actually a reasonable prediction from the perspective of fluency-theory [38]. Fluency theory has shown that the more fluently a perceiver can process a stimulus, the more positive one’s aesthetic evaluation of this stimulus becomes. If there is a Stroop compatibility effect on evaluations, it is therefore important to investigate whether this effect is caused by conflict negativity, fluency (i.e., non-conflict positivity), or both. Furthermore, until now previous research has mostly looked at the fluency and the evaluations of the exact same stimulus. If one could show that the fluency of one stimulus could also influence the evaluation of an associated stimulus, this would be an important extension of fluency theory.

### The present research

We report a set of experiments designed to study whether and how cognitive conflict associated with the Stroop task influences liking. To do so, we presented our participants with compatible and incompatible Stroop words. After the presentation of the Stroop words, participants were shown neutral pictures and were then asked to evaluate these pictures. In general, we expected that the negative affect evoked by a conflicting event would carry over to the stimulus co-occurring with that event. Specifically, we expected that the pictures that had become associated with Stroop conflict (with the incompatible Stroop words) would be liked less compared to the pictures associated with Stroop non-conflict (with the compatible Stroop words).

Furthermore, we explored the importance of different methodological parameters in the Stroop conflict task. The presented studies therefore include non-response prime paradigms (Studies 1–2) as well as response paradigms (Studies 2–4); they include paradigms involving the repeated pairing of stimuli with conflict and non-conflict (Studies 1–2) and paradigms involving single pairings of stimuli with (non)-conflict (Studies 3–4); the influence of Stroop word presentation duration was investigated (Study 1), and finally, we explored the direction of the Stroop compatibility effect on liking (Study 4). In the general discussion, we will reflect upon these variations to address the potential moderators that influence the effects of Stroop conflict on evaluations.

### A note on sample sizes

The present manuscript was the first in our lab on this topic. Therefore, we had no idea what to expect regarding effect sizes. In general, we aimed at 30–40 subjects for within-subjects comparisons, and at least double this amount for between-subjects comparisons. Because we estimated that the effect sizes in studies 3–4 would be smaller (in these studies stimuli and (non)-conflict were paired only once and the number of trials was lower) sample sizes were larger in those studies. For each result, we report the 95% upper and lower limits of the effect-sizes. Precautions were taken that participants could not register for more than one of our studies.

## Studies 1A and 1B: Initial evidence for Stroop conflict effects on offline liking

The main goal of the present studies was to replicate and extend Fritz and Dreisbach's [27] results—the influence of conflict negativity on associated stimuli—using offline explicit evaluations instead of online measures. Interestingly, Fritz and Dreisbach [39] recently showed that conflict prime duration can modulate the results of Stroop compatibility on online measures of affect. Specifically, in comparison to short Stroop presentation durations (200 and 400 ms), longer durations (800 ms) did not (or even reversed) the influence of conflict on online evaluative categorizations of co-occurring neutral stimuli. These results suggest that Stroop conflicts are evaluated automatically, and that negative affect paired with the neutral stimuli influences the online categorization of the stimuli within short time-windows. Whereas this notion may mainly apply to the situation in which participants have an active goal of evaluating each stimulus online, we do not know how presentation duration influences offline liking-ratings. In addition, the maximum duration time in the Fritz and Dreisbach [39] study was 800 ms, and hence, it remains to be seen whether even longer duration times may moderate the effect of Stroop conflict on stimulus evaluation. Therefore, Studies 1A and 1B explored whether relatively short (200 ms) and long (2000 ms) conflict presentation durations would influence offline evaluations.

In the present study participants were shown Stroop color words that were followed by the presentation of a neutral visual stimulus. Next, they evaluated the stimulus at the end of the study when quite some time had passed after they had been exposed to the Stroop conflict and accompanying neutral stimuli. We expected that participants would like the presented neutral stimulus less when it had co-occurred with the incompatible Stroop conflict words compared to when a stimulus had co-occurred with the compatible non-conflict words. Because our long exposure condition was far longer than in Fritz and Dreisbach’s study (2000 ms vs. 800 ms), our data-analytic strategy was exploratory rather than confirmatory.

### Study 1A: Method

#### Participants

Seventy-two adults from the United States participated in Study 1A (51 males; *M*_age_ = 33.79) in exchange for a small fee. Participants in all studies were recruited through Amazon.com’s Mechanical Turk service, an integrated participant recruitment and compensation system that is both diverse and reliable [40]. Studies were conducted using the online environment of Inquisit 4.0.2 [41], and were approved by Social Sciences’ Faculty Ethics Review Board at Utrecht University under file number FETC17-133.

#### Task & procedure

**Fig 1:**
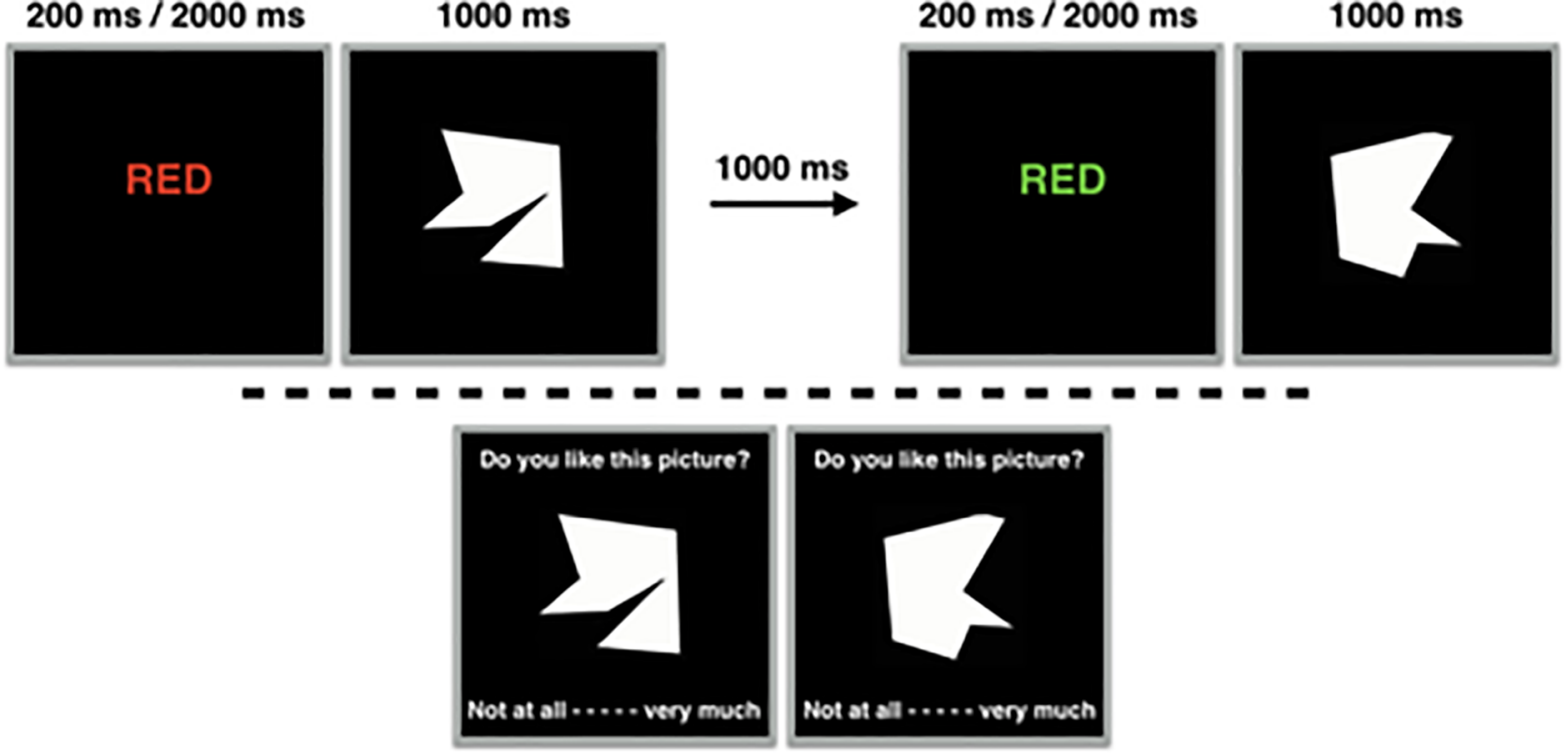
Experimental overview of Study 1A. The upper half depicts a compatible trial and an incompatible trial respectively. The lower half depicts the evaluations at the end of the study.

Participants were told that they were required to closely watch the presented stimuli, as at the end of the experiment they would be asked a number of questions about them. Participants were then presented with 32 Stroop color words (‘green’, ‘yellow’, ‘blue’, or ‘red’) in one of four colors (green, yellow, blue, or red)–there was no requirement or possibility to respond to the stimuli. In 16 trials, the Stroop color names were compatible with the colors in which they were presented, while in the other 16 trials colors and color names were incompatible. Participants were presented with the Stroop words for either for 200 or 2000 ms as a between-subjects manipulation. Immediately after being presented with a particular Stroop color word, participants were shown a neutral stimulus, which was the picture of one of two polygons randomly selected from a larger pool of polygons (selected from [42]). One of the selected polygons was always presented for 1000 ms after the compatible Stroop color words, the other polygon would always be presented after the incompatible Stroop color words. In between trials there was a delay of 1000 ms. At the end of the experiment, when participants had been shown the total of 32 Stroop-polygon pairings, participants were shown the two polygons again, one by one in random order, and were asked to indicate on a scale of 1–9 the degree to which they aesthetically liked each polygon (1 = *Extremely disliked*; 9 = *Extremely liked*). Participants were told to provide an intuitive response, and that there were no correct or wrong responses. Study 1A’s procedure is visualized in Fig 1.

### Results & discussion

A 2 (Stroop compatibility: compatible vs. incompatible) x 2 (Stroop presentation duration: 200 ms vs. 2000 ms) repeated measures Analysis of Variance (ANOVA) showed the expected compatibility effect: Polygons following incompatible Stroop words were liked less compared to polygons following compatible Stroop words (*M*_Compatible_ = 5.43, *SD* = 1.98 vs. *M*_Incompatible_ = 4.60, *SD* = 2.09; *F*(1, 70) = 6.26, *p* = .015, η^2^_p_ = .08 [upper and lower limits of effect sizes CI_95%_: η^2^_p_ = .01, .20]). More importantly, the results showed a significant interaction effect between the Stroop compatibility and Stroop presentation duration conditions, *F*(1, 70) = 4.19, *p* = .044, η^2^_p_ = .06, [CI_95%_: η^2^_p_ = .0008, .16]. Subsequent comparisons showed a strong compatibility effect when participants were presented with the Stroop words for 2000 ms (*M*_Compatible_ = 6.00, *SD* = 2.06 vs. *M*_Incompatible_ = 4.50, *SD* = 1.95; *F*(1, 31) = 8.93, *p* = .005, η^2^_p_ = .22, [CI_95%_: η^2^_p_ = .04, .40]), yet the compatibility effect disappeared when participants were presented with the words for 200 ms (*M*_Compatible_ = 4.85, *SD* = 1.88 vs. *M*_Incompatible_ = 4.70, *SD* = 2.16; *F*(1, 39) = 0.12, *p* = .730, η^2^_p_ < .01, [CI_95%_: η^2^_p_ = .00, .08]).

The results of Study 1A are clear-cut: Stroop conflict negativity influenced offline measured evaluations of stimuli accompanying conflict. In the light of Fritz and Dreisbach’s [39] findings it is interesting to note that this effect did not emerge after Stroop word presentation durations of 200 ms, but rather after much longer presentation durations of 2000 ms. Accordingly, to examine the solidness of the 2000 ms Stroop word presentation duration effect we deemed it important to test the 2000 ms presentation duration effect again.

### Study 1B: Method

#### Participants & methods

Forty-one adults from the United States participated in Study 1B (21 males; *M*_age_ = 32.73). The procedure and test setting was similar to the one described in Study 1A, with the exception that the number of trials in the compatible and incompatible conditions was increased from 16 to 24, and that all participants were presented with the Stroop words for 2000 ms.

### Results

A two-level (Stroop compatibility: compatible vs. incompatible) repeated measures ANOVA showed the expected compatibility effect: Polygons following incompatible Stroop words were liked less compared to polygons following compatible Stroop words (*M*_Compatible_ = 6.02, *SD* = 2.24 vs. *M*_Incompatible_ = 4.66, *SD* = 1.84; *F*(1, 40) = 7.26, *p* = .010, η^2^_p_ = .15, [CI_95%_: η^2^_p_ = .02, .32]). In short, the results of Study 1B replicate the findings of the 2000 ms condition in Study 1A.

### Discussion

The results from Studies 1A and 1B showed the predicted compatibility effect. Polygons were liked less when they were associated with incompatible (conflict) rather than compatible (non-conflict) Stroop words, but only when they were presented for 2000 ms, not when the words were presented for 200 ms. These results are partially in line with previous research: Studies by Dreisbach and Fischer [5] showed presentations of Stroop conflict primes generated immediate negativity (i.e., a facilitation of responding to negative targets after conflict primes). The present results add to these findings and show that conflict and conflict negativity also influence our more explicit and offline evaluations of associated stimuli.

However, while we observed that Stroop conflict negativity influenced evaluations after relatively long presentation durations (2000 ms) but not after brief presentation durations (200 ms), Fritz and Dreisbach [39] showed immediate conflict negativity to be especially strong after short durations (200 and 400 ms), yet also that this conflict negativity quickly decreased for longer (800 ms) presentation durations. The study by Fritz and Dreisbach investigated online affect, whereas we measured evaluations offline (at the end of study). When one considers both research lines it appears that in contrast to the immediate effects of conflict negativity, only prolonged exposure to conflict will influence offline and explicit evaluations. However, it is important to note that the present studies examined the effects of relatively long presentation durations (2000 ms). Hence, we do not know yet whether differences in presentation times (800 vs. 2000 ms) or differences in online vs. offline assessment of evaluation explain the empirical differences. We return to this issue in the General Discussion.

## Studies 2A – 2C: Prime versus response conflict

In Studies 1A and B we showed that stimuli were liked less when they co-occurred with incompatible Stroop words compared to when stimuli co-occurred with compatible Stroop words. It is important to note that these compatibility effects emerged without the requirement to respond to the Stroop words. This is interesting, because the original conflict model suggested that the conflict-monitoring module is activated by the concurrent activation of conflicting *response* alternatives [13], and that it is the conflict in response alternatives we experience as negative [3]. However, as we show, the negativity associated with conflict emerges even when there is no requirement to respond. These findings fit very well with a recent theoretical approach by Dreisbach and Fischer [4], suggesting that Stroop conflict is inherently negative—independent of whether it requires an immediate response.

Until we empirically compare the conflict with and without a requirement to respond, all interpretations of conflict negativity remain indirect. Therefore, to test the unique contributions of response and non-response conflict negativity we ran a number of additional studies. In Studies 2A, 2B, and 2C, participants were again presented with compatible and incompatible Stroop color words, subsequently followed by neutral stimuli. Again, we expected that stimuli following incompatible Stroop words would be liked less than stimuli following compatible Stroop words. Additionally, we explored whether compatibility-effects also emerged when participants were required to respond (Study 2A); and, whether the absence or presence of the requirement to *respond* to the Stroop words would moderate the compatibility effect (Studies 2B and 2C). In Studies 2B and 2C, participants were therefore either presented the Stroop words without being asked to respond (we refer to this as the *prime conflict task*), or participants had to indicate as quickly and accurately as possible the presented color of the word (we refer to this as the *response conflict task*; this is in fact the classical Stroop Task). The requirement to respond or not was manipulated between-subjects in Study 2B, and within-subjects in Study 2C. If the requirement to respond indeed moderates the compatibility effect on liking, we should observe a significant interaction between the Stroop compatibility and the response requirement conditions.

### Method

#### Participants

Forty-five adults from the United States participated in Study 2A (14 males; *M*_age_ = 21.69), one-hundred-and-two participated in Study 2B (66 males; *M*_age_ = 35.02), and thirty-six participated in Study 2C (23 males; *M*_age_ = 30.11) in exchange for a small fee.

#### Task & procedure

**Fig 2:**
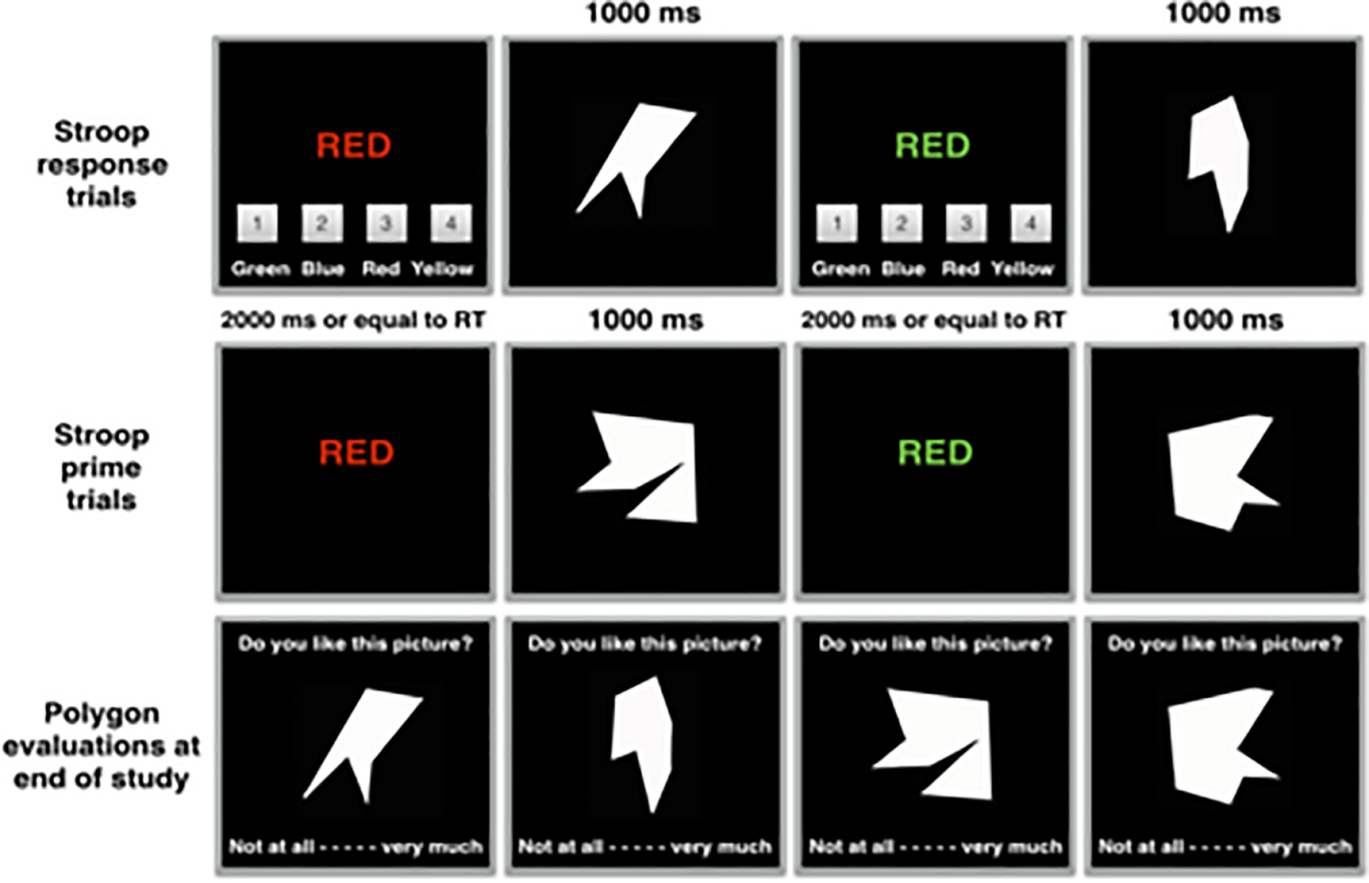
Experimental overview of studies 2B and 2C. The upper row depicts two typical compatible and incompatible trials that require a response. The middle row depicts two typical compatible and incompatible trials that do not require a response. The row below depicts the evaluations at the end of the experiment.

Study 2A was similar to Study 1B. Participants were again shown 24 compatible and 24 incompatible Stroop color words, which were paired with randomly selected polygons that were evaluated at the end of the experiment. Crucially however, is that participants were not merely presented with the Stroop color words as in Study 1B, they were also required to provide a response. Participants in this response conflict task were instructed to quickly and accurately click one of four buttons that matched the color of the Stroop word. Consequently, whereas the Stroop presentation times were presented for a fixed 2000 ms in the prime conflict task as in Study 1B, Stroop presentation times in the response conflict task in Study 2A varied and ended the moment a response was given. After a correct response, participants were presented with a neutral stimulus for 1000 ms, the picture of one of two polygons randomly selected from a larger pool of polygons. After an incorrect response no polygon was presented. In all of the response conflict studies, participants performed very accurately, erring on average on only 1% of the trials. Therefore, to provide an indication of Stroop performance we will report the results on Response Times (RTs) and not on response accuracy (though as expected, when errors did occur in the response-conflict tasks in this paper, they were most likely to be observed on incompatible trials rather than compatible trials). The procedures of the response and prime conflict tasks are visualized in Fig 2.

In Study 2B we employed a between-subject design to examine the differences between prime and response conflict. Participants either performed the prime conflict task as in Study 1B, or performed the response conflict task as in Study 2A.

In Study 2C the design was within-subjects: Participants first completed the response conflict task and evaluated the polygons that had become associated with conflict and non-conflict respectively. Participants subsequently performed the prime conflict task and evaluated different conflict and non-conflict polygons. This order allowed us to control for the word durations between the response and prime conflict tasks, such that response latencies from the response task featured as word presentation durations in the prime conflict task. One participant in Study 2C was removed from the analysis for making an excessive amount of errors (> 30% of the trials) and likely not understanding the instructions. In none of our studies did the exclusion of participants who made mistakes lead to different conclusions about the direction of effects or significance testing.

### Results Study 2A

#### Effects on liking

A repeated measures ANOVA with Stroop compatibility (compatible vs. incompatible) as a within-levels factor revealed that participants liked the polygon that was presented after the incompatible Stroop response trials significantly less compared to the polygon that was presented after the compatible Stroop response trials (*M*_Compatible_ = 5.53, *SD* = 1.63 vs. *M*_Incompatible_ = 4.84, *SD* = 1.64; *F*(1, 44) = 6.83, *p* = .012, η^2^_p_ = .13, [CI_95%_: η^2^_p_ = .02, .29]).

#### Stroop effect

For the analyses on Response Times (RTs) in the response conflict tasks in the present paper, latencies below 400 ms were set at 400, and latencies beyond 4000 ms were set at 4000. Over 99% of the response latencies occurred within these ranges. Results showed the Stroop effect (*M*_Compatible_ = 1053, *SD* = 241 vs. *M*_Incompatible_ = 1278, *SD* = 287; *F*(1, 44) = 87.39, *p* < .001, η^2^_p_ = .67, [CI_95%_: η^2^_p_ = .51, .75]).

### Results Studies 2B & 2C

#### Effects on liking

Liking scores were analyzed in a 2 (Stroop compatibility: compatible vs. incompatible) x 2 (Task: prime conflict task vs. response conflict task) repeated measures ANOVA, with Task as a between-subjects factor in Study 2B, and as a within-subjects factor in Study 2C. In both studies, there was a robust Stroop compatibility effect: Polygons following incompatible Stroop words were liked less compared to polygons following compatible Stroop words (Study 2B: *M*_Compatible_ = 5.87, *SD* = 1.85 vs. *M*_Incompatible_ = 4.63, *SD* = 1.89; *F*(1, 100) = 24.26, *p* < .001, η^2^_p_ = .20, [CI_95%_: η^2^_p_ = .09, .30]; Study 2C: *M*_Compatible_ = 5.53, *SD* = 1.54 vs. *M*_Incompatible_ = 4.67, *SD* = 1.04; *F*(1, 34) = 9.08, *p* = .005, η^2^_p_ = .21, [CI_95%_: η^2^_p_ = .02, .42];). There were no main effects of the Task conditions (Study 2B: *F*(1, 100) = 1.15, *p* = .286, η^2^_p_ = .01, [CI_95%_: η^2^_p_ = .00, .07]; Study 2C: *F*(1, 34) = 0.43, *p* = .516, η^2^_p_ = .01, [CI_95%_: η^2^_p_ = .00, .13]).

Although the results showed the main Stroop compatibility effect, the magnitude of this effect did not depend on whether participants were required to respond to or merely observed the Stroop words. In neither study did we find evidence for an interaction between the Stroop compatibility and the Task conditions (Study 2B: *F*(1, 100) = 1.68, *p* = .198, η^2^_p_ = .02, [CI_95%_: η^2^_p_ = .00, .08]; Study 2C: *F*(1, 34) = 0.52, *p* = .475, η^2^_p_ = .02, [CI_95%_: η^2^_p_ = .00, .13]) (see Table 1 for cell means). Bayesian model averaging techniques using JASP [43, 44] targeting possible interaction effects revealed BF10_Inclusion_’s of 0.462 for Study 2B and 0.203 for Study 2C, indicating no support for the interaction effects; instead they provide weak and moderate support for the null-hypothesis.

**Table 1:** Results Studies 2B & 2C.

Study 2B				Study 2C			
response conflict		prime conflict		response conflict		prime conflict	
*com-patible*	*incom-patible*	*com-patible*	*incom-patible*	*com-patible*	*incom-patible*	*com-patible*	*incom-patible*
5.56 (1.84)	4.64 (1.53)	6.18 (1.82)	4.61 (2.15)	5.51 (1.56)	4.83 (1.50)	5.54 (1.90)	4.51 (1.48)
*F*(1, 44) = 10.04, *p* = .003, η^2^_p_ = .19, [CI_95%_: η^2^_p_ = .04, .34]		*F*(1, 56) = 16.56, *p* < .001, η^2^_p_ = .23, [CI_95%_: η^2^_p_ = .08, .37]		*F*(1, 34) = 4.32, *p* = .045, η^2^_p_ = .11, [CI_95%_: η^2^_p_ = .001, .28]		*F*(1, 34) = 6.39, *p* = .016, η^2^_p_ = .16, [CI_95%_: η^2^_p_ = .02, .33]	

Means, Standard Deviations (between parentheses), and *F*-test results for liking scores in Studies 2B and 2C.

#### Stroop effect

For the analyses on Response Times (RTs) in the response conflict tasks, latencies below 400 ms were set at 400, and latencies beyond 4000 ms were set at 4000. Over 99% of the response latencies occurred within these ranges.

A two-level (Stroop compatibility: compatible vs. incompatible) repeated measures ANOVA on RTs showed that participants were slower to respond to the incompatible trials compared to the compatible trials (Study 2B: *M*_Compatible_ = 1425, *SD* = 342 vs. *M*_Incompatible_ = 1667, *SD* = 446; *F*(1, 44) = 49.79, *p* < .001, η^2^_p_ = .53, [CI_95%_: η^2^_p_ = .34, .64]; Study 2C: *M*_Compatible_ = 1472, *SD* = 363 vs. *M*_Incompatible_ = 1751, *SD* = 363; *F*(1, 34) = 112.53, *p* < .001, η^2^_p_ = .77, [CI_95%_: η^2^_p_ = .63, .83]). This replicates the classical Stroop effect [1].

#### Correlations between Stroop responses and liking

We explored whether there were relations between measures of performance/interference on the Stroop task and subsequent evaluations of the polygons. Specifically, we checked for a correlation between the average RTs and liking scores per Stroop compatibility condition. However, these analyses yielded no significant correlations (Study 2A: *r*_Compatible_ = -.17, *p* = .271; *r*_Incompatible_ = -.24, *p* = .106; Study 2B: *r*_Compatible_ = .03, *p* = .833; *r*_Incompatible_ = -.11, *p* = .482; Study 2C: *r*_Compatible_ = -.22, *p* = .202; *r*_Incompatible_ = .11, *p* = .538). Similarly there was no significant relation between the difference scores on liking and RTs (Study 2A: *r*_difference scores_ = .09, *p* = .542; Study 2B: *r*_difference scores_ = .15, *p* = .323; Study 2C: *r*_difference scores_ = -.06, *p* = .752). For the prime conflict condition of Study 2C we explored whether the averaged word presentation durations were correlated with the liking measures, but here we did not find a significant relation either (*r*_Compatible_ = -.18, *p* = .292; *r*_Incompatible_ = .05, *p* = .764; *r*_difference scores_ = .13, *p* = .454).

It is important to note that in Study 2B the prime conflict—i.e., exposure to the (in)compatible Stroop words—always lasted for 2000 ms, whereas in Study 2C it was matched to the latencies of the response to the (in)compatible Stroop words (with sample mean latencies around 1600 ms, thus lower than 2000 ms). In this light, it is interesting to note that (averaged) response latencies did not correlate with liking in compatible and incompatible Stroop word trials, suggesting that relatively short vs. long presentation duration of conflict did not matter within the confines of the variation of averaged latencies in the present study sample. This last notion is corroborated by the absence of a relation between the compatible and incompatible trials and liking in prime conflict condition of Study 2C.

### Discussion

In Studies 1A and 1B we showed that stimuli following incompatible Stroop words were liked less compared to stimuli following compatible Stroop words. In Studies 2A, 2B, and 2C we replicated this compatibility effect. Additionally, the data from the present studies suggest that *response* interference may not influence the degree of stimulus liking above and beyond the negativity experienced by the perception of conflict: There was no interaction effect between Stroop compatibility and the requirement to respond or not. Accordingly, because of the absence of an interaction effect in both (between and within-subjects designed) studies, we conclude that the compatibility effect was similar for both the response conflict and prime conflict tasks. Furthermore, the analyses did not yield significant correlations between performance on the Stroop task and the offline explicit liking ratings, suggesting that conflict experience effects on offline ratings of liking of neutral stimuli are rather insensitive to variations in duration of Stroop conflict.

Although we found no evidence for effects due to response interference, this does not mean that interference in responding does not have any part in the relation between cognitive conflict and affect. For example, Schouppe and colleagues [45] used online measures to track the relation between conflict and the speed of categorizing positive and negative words, and showed that successful responding to cognitive conflict–conflict resolution–can actually be experienced as positive. Our results however suggest that this brief positive experience does not sufficiently ‘stick’ to meaningfully influence offline and explicit evaluations.

## Studies 3A and 3B: Single prime and response conflict and effects on online liking

In the studies presented thus far participants were asked to evaluate the polygons after a number of consecutive trials. Participants had therefore been repeatedly exposed to the pairings of polygons with the compatible and incompatible trials, and produced an evaluation of the polygon *offline*. This, as has been revealed by the results thus far, led to a convincing compatibility effect. To further our understanding of the role of Stroop conflict in altering evaluations of neutral stimuli, and to increase the ability to compare our studies with previous work we now examine two key questions: (a) does prime conflict influence *online* explicit liking ratings when prime conflict duration is relatively long (2000 ms instead of the 800 ms used by [39]), and (b) do variations in response conflict durations on a trial-by-trial basis correlate with the effects of conflict on online liking?

Therefore, in the next studies, participants again performed the prime conflict task (Study 3A) or the response conflict task (Study 3B). This time, however, participants were shown a *new* (and different) polygon after every Stroop trial, which they were then required to evaluate immediately (cf. [27]). Thus, we now examine the online liking ratings of different stimuli when those stimuli are presented after Stroop (non)-conflict. Based on the previous results, overall we expected that polygons are liked less following incompatible Stroop trials relative to polygons following compatible Stroop trials.

### Method

#### Participants

Fifty-one adults from the United States (32 males; *M*_age_ = 32.71) in Study 3A, and sixty-five adults from the United States (43 males; *M*_age_ = 32.18) in Study 3B, participated in exchange for a small fee.

#### Task & procedure

Study 3A was similar to the prime conflict tasks of the previous studies, however in this study there were 16 compatible and 16 incompatible Stroop trials. Each trial featured a 2000 ms Stroop prime presentation, and a new polygon randomly selected from a pool of thirty-two polygons. Instead of being required to indicate their liking at the end of the experiment, participants had to indicate how much they liked the polygons after each trial. Four participants were removed from the analysis as their results showed they gave the same evaluative response on all trials. In none of our studies did the removal of participants for a lack of response variance lead to different conclusions about the direction of effects or significance testing. The non-excluded participants typically showed much more variance as can be observed from the standard deviations in the results section below.

Study 3B was similar to the response conflict tasks of the previous studies, and included 16 compatible and 16 incompatible Stroop trials, each trial featuring a new polygon randomly selected from a pool of thirty-two polygons. As in Study 3A, participants were required to indicate how much they liked the polygons after each trial. Four participants were removed from the analysis as they provided the same evaluative response on all trials.

### Results

#### Effects on liking

The two-level (Stroop compatibility: compatible vs. incompatible) repeated measures ANOVA in both studies showed the expected compatibility effect: The polygons, which were evaluated immediately after being presented, were liked less when paired with the compatible rather than with the incompatible Stroop words (Study 3A: *M*_Compatible_ = 4.73, *SD* = 1.23 vs. *M*_Incompatible_ = 4.45, *SD* = 1.15; *F*(1, 46) = 5.08, *p* = .029, η^2^_p_ = .10, [CI_95%_: η^2^_p_ = .006, .24]; Study 3B: *M*_Compatible_ = 4.38, *SD* = 1.04 vs. *M*_Incompatible_ = 4.16, *SD* = 0.99; *F*(1, 60) = 4.33, *p* = .042, η^2^_p_ = .07, [CI_95%_: η^2^_p_ = .001, .18]).

#### Stroop effect

The analysis on RTs in Study 3B showed the Stroop effect (*M*_Compatible_ = 1538, *SD* = 274 vs. *M*_Incompatible_ = 1723, *SD* = 331; *F*(1, 60) = 49.85, *p* < .001, η^2^_p_ = .45, [CI_95%_: η^2^_p_ = .29, .57]).

#### Relation between RTs and liking scores

The design of Study 3B allowed us to analyze the relationship between RTs and liking scores on a trial-by-trial basis. A linear mixed modelling analysis however revealed no predictive value of the RTs on the liking scores (*F’*s < 1, n.s).

### Discussion

We replicated previous findings: Again, the polygons associated with the incompatible Stroop trials were liked less compared to the polygons associated with the compatible Stroop trials. The results from Studies 3A and 3B show that compatibility effects on the explicit evaluations also occurred when measured in an online fashion. These effects materialized when the prime conflict duration was 2000 ms, or response conflict varied as a function of response latency. In short, conflict duration time did not matter much in modulating online explicit liking ratings of neutral stimuli.

## Study 4: Response conflict and the direction of effects on online liking

In the studies presented thus far we based our ideas and interpretations on one important underlying assumption: That conflicts are experienced as negative events and that the negativity associated with conflict is causing the compatibility effect on liking. However, it is also possible that the *non*-conflict words have *positive* effects on the subsequent evaluations. To explore the direction of the compatibility on liking, in Study 4 we used the same setup as Study 3B, but with the addition of control non-word trials. We expected to observe more negative evaluations after incompatible words, than after non-words and compatible words. Additionally, if the absence of conflict is experienced as a positive event, it was our expectation that we would observe more positive evaluations after the compatible Stroop words than after the non-word trials.

### Method

#### Participants

Sixty adults from the United States (38 males; *M*_age_ = 33.50) participated in exchange for a small fee.

#### Task & procedure

**Fig 3:**
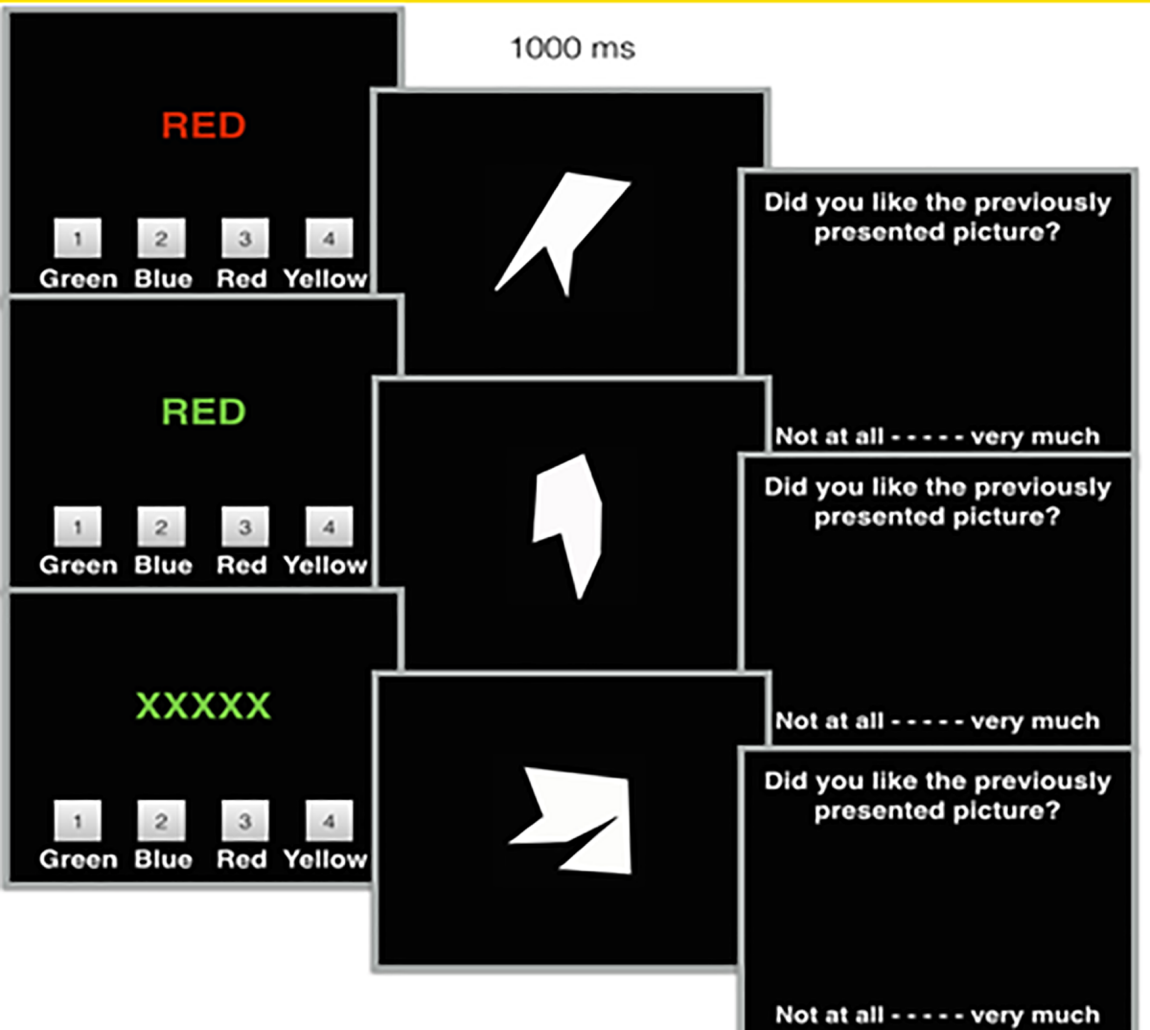
Experimental overview of Study 4.

Study 4 was similar to the response conflict setting of Study 3B, however, it featured 16 compatible and 16 incompatible Stroop-polygon pairing, as well as an additional set of 16 control trials in which a row of X-es was presented (‘XXXXX’) in the four Stroop colors. Each trial featured a new polygon, which was evaluated immediately after each Stroop trial. Study 4’s procedure is visualized in Fig 3. One participant was removed from the analysis as results showed that on all trials the same evaluative response was given. Three participants were removed from the analysis for making an excessive amount of errors (> 30% of the trials) and likely not understanding the instructions.

### Results

#### Effects on liking

A three-level (Stroop compatibility: compatible vs. control non-word vs. incompatible) repeated measures ANOVA on the liking scores showed a main effect of Stroop compatibility, *F*(2, 110) = 6.56, *p* = .002, η^2^_p_ = .11, [CI_95%_: η^2^_p_ = .02, .19]. Planned contrasts showed that incompatible trials led to lower evaluations compared to compatible trials (*M*_Compatible_ = 4.80, *SD* = .93 vs. *M*_Incompatible_ = 4.40, *SD* = .94; *F*(1, 55) = 10.22, *p* = .002, η^2^_p_ = .16, [CI_95%_: η^2^_p_ = .04, .30]). The results also suggest effects of both compatibility as well as incompatibility: Compatible words increased liking compared to control non-words (*M*_Compatible_ = 4.80, *SD* = .93 vs. *M*_Control_ = 4.63, *SD* = .87; *F*(1, 55) = 5.01, *p* = .029, η^2^_p_ = .08, [CI_95%_:η^2^_p_ = .005, .21]), and incompatible response words (marginally) reduced liking compared to the control non-words (*M*_Inompatible_ = 4.40, *SD* = .94 vs. *M*_Control_ = 4.63, *SD* = .87; *F*(1, 55) = 3.47, *p* = .068, η^2^_p_ = .06, [CI_95%_: η^2^_p_ = .00, .18]).

#### Stroop effects

In the similar analysis on RTs, there was a main effect of Stroop compatibility, *F*(2, 110) = 51.94, *p* < .001, η^2^_p_ = .49, [CI_95%_: η^2^_p_ = .36, .56]. Planned contrasts showed that incompatible Stroop words led to higher RTs compared to compatible Stroop words (*M*_Compatible_ = 1234, *SD* = 310 vs. *M*_Incompatible_ = 1433, *SD* = 364; *F*(1, 55) = 95.04, *p* < .001, η^2^_p_ = .63, [CI_95%_: η^2^_p_ = .50, .72]). RTs for the control non-words were lower than for the incompatible Stroop words (*M*_Incompatible_ = 1433, *SD* = 364 vs. *M*_Control_ = 1242, *SD* = 304; *F*(1, 55) = 54.28, *p* < .001, η^2^_p_ = .50, [CI_95%_: η^2^_p_ = .33, .61]), there was however no difference between the RTs for the control non-words and for the compatible words (*M*_Compatible_ = 1234, *SD* = 310 vs. *M*_Control_ = 1242, *SD* = 304; *F* < 1, n.s.).

#### Relation between RTs and liking scores

A linear mixed modelling analysis indicated that RTs had no predictive value for the liking scores in the compatible condition (*F*(1, 892) = 0.61, *p* = .436), nor in the control condition (*F*(1, 893) = 2.12, *p* = .145). It did reveal a marginal effect of RTs on liking scores in the incompatible condition (*F*(1, 880) = 2.78, *p* = .096). However, given the considerable power of the linear mixed modelling method, and given the just marginally significant effect, we believe it is justified to conclude that RTs had no predictive value for the liking scores in the incompatible condition.

### Discussion

We replicated the main compatibility effect as shown in the previous studies. Again the stimuli associated with the incompatible Stroop words were liked less compared to the stimuli associated with the compatible Stroop words. However, the addition of a control condition enabled us to investigate the direction of the compatibility effect. The significant difference between the compatible and control trials is noteworthy, as it also indicates a positive influence of the compatible words. We will discuss these results more thoroughly in the General Discussion.

## Meta-analysis

To provide a statistically robust estimate of the effect size of the compatibility effect we conducted a meta-analysis by synthesizing the data from all reported studies (including studies reported in footnotes). Additionally, we wanted to explore the nature and boundary conditions of the compatibility effect further by testing potential moderators of this effect. In some studies we induced prime conflict (Study 1A, Study 1B, Study 3A), in other studies we induced response conflict (Study 2A, Study 3B, Study 4), and in other studies we contrasted both types of conflict (Study 2B, Study 2C). Although type of conflict was not a significant moderator of the compatibility effect in Study 2B and Study 2C, it is possible that a more powerful meta-analysis across all studies reveals a significant moderation effect. In some studies stimuli were paired repeatedly with (non)-conflict, and we assessed evaluations offline at the end of the study (Study 1A, Study 1B, Study 2A, Study 2B, Study 2C), and in other studies we assessed them online after a single pairing (Study 3A, Study 3B, Study 4). In the meta-analysis, we investigated whether the type of conflict and whether effects were measured offline or online (and were paired repeatedly or only once) moderated the compatibility effect.

### Included in the meta-analysis: Replications of 3A & 3B

We report the results of one replication study that was virtually identical to Study 3A (perceptual conflict) and two replication studies that were virtually identical to Study 3B (response conflict).

The sample (*N* = 33) recruited to replicate of Study 3A showed the same compatibility effect on liking (*M*_Compatible_ = 4.89, *SD* = 1.08 vs. *M*_Incompatible_ = 4.45, *SD* = 1.20; *F*(1, 32) = 8.07, *p* = .008, η^2^_p_ = .20, [CI_95%_: η^2^_p_ = .03, .38]). Note that a small programming error led to unequal trial distribution over the experimental conditions in this study.

The first sample (*N* = 51) pertaining to the replication of Study 3B showed the same compatibility effect on liking (*M*_Compatible_ = 4.91, *SD* = 1.09 vs. *M*_Incompatible_ = 4.56, *SD* = 1.01; *F*(1, 50) = 8.89, *p* = .004, η^2^_p_ = .15, [CI_95%_: η^2^_p_ = .03, .30]), and the same the Stroop effect (*M*_Compatible_ = 1242, *SD* = 281 vs. *M*_Incompatible_ = 1515, *SD* = 370; *F*(1, 50) = 72.40, *p* < .001, η^2^_p_ = .59, [CI_95%_: η^2^_p_ = .43, .69]).

The other sample (*N* = 42) aimed at replicating Study 3B showed the Stroop effect (*M*_Compatible_ = 1621, *SD* = 301 vs. *M*_Incompatible_ = 1855, *SD* = 408; *F*(1, 41) = 34.90, *p* < .001, η^2^_p_ = .46, [CI_95%_: η^2^_p_ = .26, 0.59]), but did not show the compatibility effect on liking, *F*(1, 41) = 1.09, *p* = .303, η^2^_p_ = .03, [CI_95%_: η^2^_p_ = .00, .15].

### Meta-analysis method

We computed effect sizes and conducted the moderator analyses with the aid of a computer program [46]. To calculate effect sizes we entered the *M*s, *SD*s, and *N*s of the liking scores related to compatible and incompatible trials from all studies. Because the compatibility effect is a within-participants effect, we also entered the correlations between the liking scores related to the compatible and incompatible trials [47]. As type of conflict was experimentally manipulated between participants in a single study (Study 2B), we used between-participants conditions as the unit of analysis. We did not enter the compatible and incompatible blocks of Study 2B as separate effect sizes because they were administered within-participants, and hence, this would have violated the independence of effect sizes in the meta-analysis [48]. This yielded *k* = 13 independent effect sizes available for the meta-analysis. Because the compatibility effect involves a comparison between two means, we chose Cohen’s *d* as the effect size statistic. According to convention [49], the magnitude of the effect size can be interpreted as: small (0.2); medium (0.5); large (0.8).

For the overall analysis and the moderator analyses we chose a random-effects model. The random effects model assumes that there is variation in effect sizes beyond sampling error that can be attributed to systematic factors (i.e., moderators). This model was advocated over a fixed-effects model, which posits that all variations in effect sizes are attributable only to sampling error. Given the methodological differences between the studies and between-participants conditions, the assumption of a fixed-effects model was likely violated, and therefore a random-effects model should be advocated (e.g., [47]). Following meta-analytic conventions, however, we formally tested whether there are indeed systematic differences between the effect sizes. We did this by calculating the within-class goodness-of-fit statistic Q_W,_ which is approximately chi-square distributed, with *df* = *k*—1 (where *k* is the number of effect sizes). The heterogeneity test was highly significant, Q_W_(12) = 38.43, *p* < .001, indicating that the effect sizes indeed systematically differed from each other. The I^2^ statistic [50] indicated that 69% of the variance in effect sizes was attributable to systematic differences between effect sizes. This result indicates that the larger share of the variance in effect sizes can be attributed to systematic factors rather than to sampling error. This justifies the choice for a random-effects model, and also implies that a moderator analysis is sensible.

### Results

#### Overall effect size

The random effects model yielded a mean estimated effect size of *d* = .364, *SE* = .076, with a 95% confidence interval of .216 - .512. The effect was significantly different from zero, *Z* = 4.816, *p* < .001. Based on this result we can safely conclude that the compatibility effect is a genuine experimental effect, and that it has a small to medium effect size [49].

#### Moderator analysis

We used the categorical model test [51] to analyze the potential moderating role of type of conflict and the number of times stimuli had been associated with conflict and non-conflict. We calculated the between-class goodness of-fit statistic Q_B_, with *df* = *j*—1 (where *j* is the number of categories per moderator). A large and significant Q_B_ indicates that the variability in effect-sizes is at least partially explained by the moderator. Thus, Q_B_ is roughly similar to a main effect in an ANOVA.

Study 2C was omitted from the moderator analysis of type of conflict because in this study all participants experienced prime conflict and response conflict. The analysis showed that type of conflict did not significantly moderate the compatibility effect, Q_B_(2) = 2.28, *p* = .356. The compatibility effect was significant irrespective of whether it was operationalized as a prime conflict (*Z* = 3.86, *p* < .001) or as a response conflict (*Z* = 2.70, *p* = .007). Thus, the effect size related to prime conflict was not statistically larger than the effect size related to response conflict, *d* = .426, [CI_95%_: .210, .642] and *d* = .285, [CI_95%_: .078, .492], respectively.

In contrast, the mode of measurement and number of pairings between stimuli with (non)-conflict (offline/repeated pairings vs. online/single pairings) was a significant moderator of the compatibility effect, Q_B_(2) = 4.15, *p* = .042. Although the compatibility effect was significant irrespective of whether evaluations of stimuli were measured offline after repeated pairings (*Z* = 4.89, *p* < .001) or online after single pairings (*Z* = 2.71, *p* = .007), the effect size was significantly larger for offline measures with repeated pairings relative to the effect size for online measurements with single pairings, *d* = .512, [CI_95%_: .307, .717] and *d* = .235, [CI_95%_: .065, .405], respectively.

### Discussion

The meta-analysis showed that across studies the compatibility effect was highly significant and has a small to medium effect size, *d* = .364. To compare, a recent meta-analysis across 214 studies of the evaluative conditioning effect (which may resemble the effects of conflict-evoked negativity on evaluations examined in the present studies) found a mean estimated effect size of *d* = .524 [34]. The compatibility effect thus seems to be smaller than the average evaluative conditioning effect. However, evaluative conditioning studies typically measure offline evaluations at the end of the study after repeated pairing between the UC and CS. Interestingly, the current meta-analysis showed that when evaluations were assessed offline and stimuli had been repeatedly paired with (non)-conflict, the effect size of the compatibility effect was similar to the estimated mean effect size established by Hofmann and colleagues (*d* = .512) [34].

Type of conflict did not moderate the compatibility effect. Whether or not participants were required to respond to the Stroop words did not affect the magnitude of the effect. These results do not lend support to the original conflict model that suggested that the negative experience is mainly caused by response conflict [13]. The mere priming of cognitive conflict appears to be enough to activate negativity that, in turn, influences evaluations of related stimuli.

A limitation of the moderator analysis was that we could not control for potential confounds between experimental conditions. The six effect sizes related to offline measurement resulted from single stimulus/(non)-conflict repeated pairings, and four (67%) involved prime conflict, whereas only two (33%) involved response conflict. Reversely, the six effect sizes related to online measurement followed from different stimuli/(non)-conflict single pairings, of which four (67%) involved response conflict, whereas only two (33%) involved prime conflict. To assess the independent contribution of each moderator, a simultaneous inclusion of the two moderators in one and the same analysis would be desirable. This procedure is notoriously difficult in a meta-analysis, and may better be accomplished in future experimental studies.

## General discussion

The present research investigated whether conflict negativity influences explicit evaluations of associated stimuli. In most of our studies we found that stimuli co-occurring with Stroop conflict were liked less compared to stimuli that did not co-occur with Stroop conflict.

### The conflict negativity hypothesis

The present findings are generally in line with earlier findings showing that conflict triggers brain areas associated with error detection [23], with negativity [22][24], and with the speeded-up detection of negative targets [27]. These results are also in line with Fritz and Dreisbach’s [3] and [39] approach to cognitive conflict as an inherently negative signal. Our results lend support to these findings by showing that cognitive conflict may not only elicit an immediate affective reaction, but on the longer term can also influence people’s explicit evaluations and attitudes.

### Response and prime conflict

The present research supports the notion that conflict negativity emerges through the activation of conflicting cognitive processes, even without a requirement to respond. This was already suggested by previous studies (e.g., [27]) but the current research directly manipulated and investigated the difference in conflict negativity between response and prime conflict.

We also investigated whether response interference would influence evaluations above and beyond the effect of prime conflict. It was actually response interference that was originally assumed to be the key process underlying cognitive conflict ([13], see also [20]). However, we found no strong evidence for any additional effects attributable to interference in responding, as the effects between prime conflict and response conflict were similar. This does not *prove* that *response* interference or successful conflict resolution have no affective consequences (see for example [45])—we actually consider it likely that they do influence affective responses. However, what our results minimally show is that the response component does not meaningfully influence explicitly generated liking ratings and evaluations. An interesting avenue for future research is therefore to track the relation between conflict (resolution) over time using both measures of immediate/automatic affect, as well as offline explicit measures.

### Effects similar to evaluative conditioning may emerge out of conflict negativity

The present research shows that effects similar to Evaluative Conditioning (e.g., [33]) occur using unconditioned stimuli that are neutral in valence (i.e. meaning), but that nevertheless trigger negativity through their conflicting features. This is relevant because of the importance of EC-theory as a model for human cognition and behavior. EC-theory explains how people develop likes and dislikes for objects, and how they learn to respectively approach or avoid those objects. Our results suggest that such (dis)likes develop not only through associations with objects that are positive and negative in meaning, but also when neutral stimuli trigger processing conflicts through their perceptual features.

#### Degree of conflict and affect

Note that we did not find evidence for a relation between the *degree* of conflict and any changes in liking. Although the degree of conflict may not have been accurately captured using RTs (e.g., [52]), we do not wish to completely rule out other accounts that could explain why the Stroop task causes changes in liking: Individuals may have noticed some kind of wrongness or incongruency that determined how individuals evaluated associated items–perhaps independently of any cognitive conflict (e.g., [53][54][55][56]). In other words, participants may have become conscious of the repeated associations between certain polygons and conflict and non-conflict respectively. Given the absence of other information, such specific awareness may have led them to recode difficult trials as something to be disliked and easy trials as something to be liked. As such, the influence of contingency awareness on the present effects is something future research should address. Therefore, while previous studies have shown that both response conflict (e.g., [19][21]) as well as ‘perceptual’ conflict (e.g., [5][27]) automatically triggers negative affect, the support for a relation between conflict and conflict-negativity remains tentative. Other measures of conflict than RTs, implicit manipulations, and manipulations of conflict-stimulus contingencies therefore seem promising avenues for future research.

### The positivity of non-conflict and fluency

Building on literature on conflict negativity (e.g., [3][4] we explicitly assumed that the effects of Stroop compatibility on liking would be caused by the conflicting incompatible Stroop words. However, we showed in Study 4 –a study featuring control trials–that while incompatible words indeed reduced liking, compatible words simultaneously increased liking. Before jumping to any conclusions based on the results of Study 4, it is important to remember that we only had such a control condition in one single study that featured specific methodological parameters–response task, single pairing of stimuli with (non)-conflict, ‘online’ measures–and that it is unclear whether the same effects would occur when using the non-response priming task, or when using the repeated pairings design. Furthermore, it is still tentative whether the control trials that were used (the row of X-es) represent a ‘true’ baseline.

Such considerations aside, we do wish to speculate on some of the reasons that may underlie the positivity associated with the compatible non-conflict Stroop words. First of all, because conflict and non-conflict occurred within participants it could be that in the context of conflict and conflict negativity, a non-conflicting stimulus may evoke positive affect. In other words, because cognitive conflict is experienced as a negative signal, and because that conflict does not always occur, there is a cognitive ‘sense of relief’–leading to a positive affective reaction.

Apart from the negativity and positivity that may result from conflict and non-conflict, another possibility is that the compatible non-conflict words themselves evoke positive affect–without necessarily relating to the experience of conflict. A mechanism reported in the literature showing just such an effect is perceptual fluency [57]. The research on fluency has shown that the more fluently a perceiver can process an object, the more positive his or her aesthetic response is [38][58]. In the present research, a perceptual fluency on compatible trials in the Stroop task may have positively influenced subsequent evaluations. Such a finding would expand the existing models on fluency theory by showing that not only are fluent stimuli themselves liked more, but also that the fluency effect of one stimulus can carry-over, to influence the evaluations of an associated stimulus. Whether fluency indeed positively influenced evaluations, and, whether conflict and fluency are actually wholly different processes or endpoints of a gradient scale, would be something future research should establish.

### Conflict presentation times

An interesting finding in the present studies was that the observed effect seemed to be modulated by the Stroop word presentation durations. The compatibility effect was generally observed when Stroop words were presented for relatively long periods of time (2000 ms), but not when words were presented for a brief time (only 200 ms). This appears to be in contrast with results on an evaluative categorization measure reported by Fritz and Dreisbach [39]. They showed that a negativity bias towards the categorization of neutral stimuli emerged after individuals were presented with conflict stimuli for 200 and 400 ms, yet also that this bias quickly decreased (and could even reverse) for longer conflict presentation durations (800 ms).

How can we reconcile these seemingly different findings? One possibility lies in using different setups and measures. Specifically, the study by Fritz and Dreisbach [39] likely addressed affective priming effects, and as such provided an appropriate online measure to examine automatic processes (e.g., priming) and more controlled processes (e.g., affective counter-regulation) of the immediate consequences of conflict and non-conflict. In other words, it could be that effects related to affective priming occurred relatively quickly after conflict presentation, but vanished or even reversed with longer presentation duration which allowed participants to rely on a more controlled processing mode that opposed automatic affective priming effects (cf. [59]). In the present set of studies, however, we assessed the potential negativity effects of conflict on explicit liking ratings, in which there might be less room for quick and automatic affective priming effects. That is, participants did not rely on the (short-lived negative) affective primes when generating explicit evaluations of the neutral stimuli, but instead, based their evaluations on the pairings between the (relatively long-lived negative) conflicting events and the neutral stimuli. Future studies should test these theories.

Although we cannot empirically address the explanation of the different findings with respect to conflict duration, our findings at least suggest that prolonged exposure to cognitive conflict may influence evaluative processes above and beyond affective priming, such as processes related to explicit evaluations. In so doing, our findings fit well with research on evaluative conditioning, also demonstrating persuasive effects on liking when the duration to negative stimuli (unconditioned stimuli, US) is relatively long and people are not urged to respond fast, but can think about their liking of the neutral conditioned stimuli paired with the US [34].

### Stroop conflict: A potential new avenue of attitude change

The domain of attitude formation and change has always been a prime area of scientific inquiry, and due to the considerable attention devoted to this topic the powerful effects of techniques such as conditioning [60], affective priming [61], cognitive dissonance [62], heuristic processing [30][31][32], and mere exposure [63] have become parts of basic scientific knowledge. The present research shows that a compatibility—or incompatibility—among the features of a given stimulus will influence people’s evaluations of an associated stimulus: a new and important finding that potentially establishes a new avenue in the domain of attitude formation and change.

## Conclusion

In the present research we investigated the evaluative consequences of the perception of conflicting and non-conflicting stimuli in the Stroop task. This allowed us, to some degree, to investigate the principles underlying cognitive conflict, while we simultaneously explored a novel way to influence attitudes. The findings contribute to the literature by showing not only that negative associations are triggered by conflicting stimuli, but also that positive associations are triggered by non-conflicting stimuli. Whether Stroop compatibility is also able to influence pre-existing attitudes, product evaluations and consumer choices seem promising avenues to explore in future research.

Conflict experiences are omnipresent and pervasive in everyday human life. The activation of a cluster of cognitions may at one moment present a perfectly aligned picture, and at other times cognitions may be less aligned, or even be completely incompatible with one another. In the present research we show that such compatibility and incompatibility can influence evaluations, and therefore, that conflict and non-conflict emerging from the Stroop task may prove to be a powerful new tool to influence evaluations.
